# Editorial: Antimycobacterial drug discovery: Molecular therapeutics and target identification

**DOI:** 10.3389/fphar.2022.997618

**Published:** 2022-08-29

**Authors:** Hendra Gunosewoyo, Gert Kruger

**Affiliations:** ^1^ Curtin Medical School, Faculty of Health Sciences, Curtin University, Perth, WA, Australia; ^2^ University of KwaZulu-Natal, Durban, South Africa

**Keywords:** mycobacterium, tuberculosis, *Mycobacterium abscessus*, synergistic effects, drug repurposing

The genus of mycobacteria includes the causative agent of tuberculosis (*Mycobacterium tuberculosis; M. tb*) and more recently, the non-tubercular species such as *M. abscessus and M. avium*. Clinically, tuberculosis stands as the number two infectious disease killer after COVID-19 and *M. abscessus* is becoming a more prevalent pathogen among the patients with cystic fibrosis. From the basic science perspective, investigation of molecular mechanisms that these mycobacteria use to stay dormant and evade our immune system, as well as identification of novel drug targets/scaffolds, have been and will continue to be crucial in combating the infections. We are delighted to publish this Research Topic on “*Antimycobacterial Drug Discovery: Molecular Therapeutics and Target Identification*” that highlights some of the more recent contributions in this research field.

The Research Topic was launched with an original article from Falkinham III group (Virginia Tech Center, United States) describing target identification efforts for a metal-amino acid complex antibiotic against *M. smegmatis* (Karpin et al.). The authors reported the isolation of spontaneous *M. smegmatis* mutants strain mc^2^155 that were resistant to iridium-L-phenylglycine antibiotic complex. The discovery that these mutant strains were also resistant to clarithromycin guided the isolation and DNA sequencing efforts that ultimately resulted in the identification of the peptidyl transferase domain of the mycobacterial 23S rRNA as one possible target of the iridium-amino acid complex.

Quang and Jang (Gyeongsang National University, South Korea) review the current drugs and drug candidates in preclinical trials against *M. abscessus*. The drugs were classified according to their general chemical classes such as macrolides, beta-lactams, tetracyclines, oxazolidinones, and fluoroquinolones. For the more recent drug candidates, potential molecular targets and their shortcomings were also discussed where relevant, e.g., inhibition of MmpL3 by indole-2-carboxamides. This Review will be valuable for the identification of novel drugs/targets for treatment of *M. abscessus*.

Chen et al. (National Health Research Institutes, Taiwan) report the synthesis and biological studies of silver nanoparticles as antitubercular agents. The alginate-capped silver nanoparticles were characterised for their size and morphology, prior to testing them *in vitro* for their bactericidal abilities. The authors found that their silver nanoparticles were effective against both drug-resistant and dormant *M. tb* as well as in a macrophage infection model. The *in vivo* mouse TB model results were also indicative of reduced bacterial burden in lungs without significant gross toxicity.

Li et al. (Peking University Third Hospital, China) describe the ability of nitazoxanide, a broad-spectrum anti-infective agent, to inhibit RANKL-induced osteoclastogenesis. Nitazoxanide was initially known for their antiprotozoal properties and there were instances of their ability to inhibit *M. tb* proliferation. The authors found that nitazoxanide also inhibits STAT3 pathway that could be exploited in the RANKL-mediated osteoclastogenesis. Using ovariectomised-induced bone loss model, the compound was shown to ameliorate bone loss *in vivo* accompanied with suppression of transcription factor NFATc1.

Ahmed et al. (Infectious Disease Research Institute, Seattle) share their high-content screening of 10,000 small molecules against *M. tb* within macrophage-like cells. This is of clinical relevance as *M. tb* stays in an intracellular environment prior to transitioning into an active form. Five chemotypes were identified: benzene-amide ethers, phenylthioureas, phenyl pyrazoles, thiophene carboxamides and thienopyridines. Although the mechanisms of action and drug targets are yet to be identified, these scaffolds serve as useful starting points to yield antitubercular compounds that could be actively killing intracellular *M. tb* rather than the active form.

Lastly, Bich Hanh et al. (Gyeongsang National University, South Korea) perform synergistic assay in *M. abscessus* to determine which antibiotic has the optimal effects when combined with the first-line treatment clarithromycin. Amongst the antibiotics tested, omadacycline was found to be the most synergistic using an *in vitro* checkerboard combination assay. This combination regime was then tested in zebrafish model infected with *M. abscessus*, where bacterial burden reduction was observed alongside significant lifespan extension of the infected zebrafish. Future testing in rodents model would be beneficial to ascertain the synergism of clarithromycin-omadacycline combination.

Overall, the first volume this Research Topic: “*Antimycobacterial Drug Discovery: Molecular Therapeutics and Target Identification*” highlights recent advances in this field, ranging from tubercular to non-tubercular mycobacteria. We acknowledge the highly valuable contributions from all authors to make this Research Topic possible. We look forward to the future submissions for the Volume 2 of this Research Topic in the future.

